# Defining the microbial effluxome in the content of the host-microbiome interaction

**DOI:** 10.3389/fphar.2015.00031

**Published:** 2015-02-19

**Authors:** Anastasios Ioannidis, Maria Magana, Cristian G. Bologa, Tudor I. Oprea, Ian T. Paulsen, George P. Tegos

**Affiliations:** ^1^Department of Nursing, Faculty of Human Movement and Quality of Life Sciences, University of PeloponneseSparta, Greece; ^2^Department of Clinical Microbiology, Athens Medical School, Aeginition HospitalAthens, Greece; ^3^Translational Informatics Division, Department of Internal Medicine, University of New Mexico Health Sciences CenterAlbuquerque, NM, USA; ^4^Department of Systems Biology, Center for Biological Sequence Analysis, Technical University of DenmarkLyngby, Denmark; ^5^Department of Chemistry and Biomolecular Sciences, Macquarie UniversityNSW, Australia; ^6^Torrey Pines Institute for Molecular Studies, Port St. LucieFL, USA; ^7^Department of Dermatology, Harvard Medical SchoolBoston, MA, USA; ^8^Wellman Center for Photomedicine, Massachusetts General HospitalBoston MA, USA

**Keywords:** efflux transporters, multidrug resistance, microbiome, host-pathogen interaction, effluxome

## The clinical challenge: multi-antibiotic resistance is not a single event

Antibiotic resistance is recognized globally as an emerging threat for public health. The recent World Health Organization report identifies an array of multidrug resistant bacterial pathogens as emerging mortality threats (WHO, 2014). Antibiotic resistance is not the result of a single event but the unavoidable outcome of the vicious evolutionary race between the pathogens and the host. Humans have been developing and abusing generations of potent antibiotics while pathogens have been orienting their genetic arsenal toward developing an effective and adaptable resistance network. Bacterial resistance can be conventionally classified in three interactive layers: (1) the intrinsic, that includes alterations in the metabolic pathways and the “classic” antibiotic determinants (permeability barrier and efflux, inactivation and modification of genes, and pathways), (2) the acquired, which appears through mutations and horizontal gene transfer involving antibiotic determinants, and (3) the phenotypic, where metabolic pathway changes, often in response to environmental signals, lead to an altered physiology contributing to resistant phenotypes (biofilms, persister cells, quorum sensing).

Multidrug efflux systems are membrane transport proteins placed at the epicenter of intrinsic resistance and have been at the forefront of research for the last 20 years. They perform essential roles in cellular metabolism, and differ in membrane topology, energy coupling mechanisms and substrate specificities (Dean et al., 2001; Rees et al., 2009; Fletcher et al., 2010). Among their potential roles, efflux pumps are demonstrated to be important for detoxification processes of intracellular metabolites, bacterial virulence in both animal and plant hosts, cell homeostasis and intercellular signal trafficking (Martinez et al., 2009).

Based on their sequence similarity and structural homology, efflux systems are classified into six super-families (Dean et al., 2001). ATP-Binding Cassettes (ABC), Major Facilitators (MFS), Resistance-Nodulation cell Division (RND), Small Multidrug Resistance (SMR), Multi-Antimicrobial Extrusion protein family (MATE), and Multidrug Endosomal Transporters (MET). The first five families are found mainly in microorganisms, while the MET family appears restricted to higher eukaryotes. Recently, unbiased transcriptomic analyses of the *Acinetobacter baumannii* response to chlorhexidine identified a hypothetical protein as a new class of drug efflux system (Hassan et al., 2013). The clinical role of efflux systems is the subject of intensive research in emerging threats such as Methicillin-resistant *Staphylococcus aureus* (MRSA) (Lemaire et al., 2007), Gram-negative pan-drug resistant bacteria (Anyanful et al., 2005; Browning et al., 2013; Merkx-Jacques et al., 2013) and *Mycobacterium tuberculosis* (Holzinger et al., 2012).

This opinion article emphasizes the contribution of efflux to multi-antibiotic resistance, highlights examples where efflux systems are shaping host-pathogen interactions in challenging clinical conditions, comments on the advances in the discovery path of microbial efflux inhibition, and underlines the need for highly informative and comprehensive translational antimicrobial therapeutic interventions.

## The gap in translation

Most efflux target based discovery efforts have severely underestimated the dynamic nature and phenotypic complexity of microbial communities in infection sites. The microbial flora analysis in clinical human samples is informative for the importance of *microbiome* in health and disease and for the design of host oriented anti-infective approaches as well as faster and accurate outbreak diagnostic countermeasures (Peterson et al., 2009; Kraal et al., 2014). The cooperative interaction between microbial populations has a demonstrated amplification effect in multi-antibiotic resistance development in pathogens (Zhang et al., 2011), which is consistent with the notion that pathogenic microbial subpopulations are not operating independently but as members of a poly-microbial biological network. Although the resistance mechanisms have been largely studied at concentrations above the minimum inhibitory concentration (MIC), there is evidence that when antibiotics (i.e., lantibiotics) interfere with quorum sensing lead to altered virulence expression of the pathogens (Andersson and Hughes, 2014). This network is directly affected by efflux with roles that have not been clearly determined.

The human gut is a classic example where the overall functionality, expression levels, and physiological role of efflux systems remain an unexplored puzzle. Metagenomic investigations of the human gut microbiome provide individual-specific strain patterns for drug uptake and hold promise for the development of cross-referenced metagenomic databases including efflux system (Schloissnig et al., 2013). There are few examples of microbiome reference species with fully described efflux systems; among the most prominent are the RNDs in *Bacteroides fragilis* (Wexler, 2012). Scattered reports are implicating tetracycline resistance efflux systems in *Clostridium saccharolyticum* as a response to antibiotic challenge combined with ribosome protection-type resistance (Kazimierczak et al., 2008). The prevalence of tetracycline resistance loci has also been detected in honeybee gut metagenomes (Tian et al., 2012) and on swine intestinal *viriome* (phage metagenomes) (Allen et al., 2011).

Dormant persister cells and other factors contributing to antibiotic tolerance present an intriguing example for the necessity of system level approaches that will guide discovery efforts (Tan et al., 2007; Schneider and Ayres, 2008). Persisters are a cell subpopulation contributing to resistance phenomena in recurrent and chronic infections by escaping bactericidal antibiotic challenge and host immune responses (Cohen et al., 2013; Willenborg et al., 2014). It is worth mentioning that efflux system induction through oxidative stress (i.e., *E. coli*, RND AcrAB-TolC) leads to increased numbers of multidrug-tolerant persisters (Wu et al., 2012). Conversely, metabolic signals facilitate antibiotic uptake through proton-motive force generation thus stimulating persister cell killing (Allison et al., 2011).

Those observations lay the ground for a new discovery era but need to be aligned with the information linking microbiome with drug interactions and should be translated in association with host tissues and immunity. Although the majority of antibiotics used in clinical practice are well tolerated and generally safe, some of the adverse effects experienced by a small fraction of patients may be life-threatening (Dancer, 2004). The microbiota coevolves with the host and can affect its physiology and metabolism. In fact, it is suggested to function as an auxiliary, virtual organ that cooperates with the host through modulation of metabolic pathways leading to host side effects (Cryan and Dinan, 2012). The gastrointestinal antibiotic side effects represent the most frequent disturbances due to the toxicity on the extremely diverse bowel flora (Cunha, 2001).

Host tissue effects or systemic adaptive and innate immunity triggers are harder to map and detect. Treatment with antibiotics results in reduced diversity of the human microbiome. It is suggested that the adverse effects (blood dyscrasias, events in the central nervous system, drug-induced fever, arrhythmias and electrolyte disorders) are triggered by the host microbiota (Zeissig and Blumberg, 2014). Intriguingly, the gut microbiota and probiotic agents act on the levels of circulating cytokines also affecting brain function, through endocrine (catecholamines) and immune (cytokines) pathways known to participate in the brain-microbiota interplay. Additionally, the gut microbiota is strongly implicated in the hypothalamus–pituitary–adrenal axis which is regulated by cortisol secretion leading to the activation of the immune cells (Cryan and Dinan, 2012).

A microbial efflux specific example in direct connection with the immune system is related with antimicrobial peptides (AMPs). AMPs are an integral part of the innate immune system protecting a host from invading pathogens (Brogden, 2005; Nguyen et al., 2011). Cationic AMPs are considered as alternatives for antibiotics due to their broad antimicrobial activity. Antitumor activity has also been reported for AMPs (Reddy et al., 2004; Hoskin and Ramamoorthy, 2008). Both ABCs and RNDs have been associated with resistance mechanisms to AMPs. It is suggested that ABCs are importing AMPs whereas RNDs are exporting them (Nikaido, 1996; Guilhelmelli et al., 2013) but a clear pattern of this involvement has not been demonstrated.

## Target-based microbial efflux inhibition

The discovery of small molecule Efflux Pump Inhibitors (EPIs) has been a rapidly expanding discipline. Conventional wisdom, the available discovery tools and the clinical necessity for alternative therapeutic strategies have pinpointed specific transporter families as targets for efflux inhibition. The most classic examples include the RNDs in Gram-negative bacteria and the prominent ABCs in pathogenic fungi and cancer cells. There is substantial progress in the identification of lead chemotypes with EPI properties, but the inherited transporter promiscuity requires an informative translational strategy to define the principles of the interaction with the host. The pivotal role of efflux systems has been shown by advances in cell physiology and host-based transporter oriented studies. Recent identification of transporters with designated novel roles directly implicated in pathogenicity and cancer is reshaping conventional views and approaches for efflux inhibition. Several promising narrow- and broad-spectrum microbial EPIs have been characterized, but they did not result in a clinically useful countermeasure (Lomovskaya and Bostian, 2006; Kourtesi et al., 2013). In few occasions, molecules that enhance antibiotic activity and reduce *in vitro* resistance have been identified in successful preclinical development studies (Hirakata et al., 2009), hence there are currently three generations of inhibitors in mammalian systems that have failed in different stages of the clinical development pipeline (Palmeira et al., 2012).

The EPI development path may be hindered by the manipulation of efflux systems which can cause unexpected toxicity due to the multitude of physiological roles transporters play in human cells. Target bacteria seem to respond to clinical challenge with EPIs through decreasing their efficacy by developing resistance mutations (Ahmed et al., 1993; Klyachko et al., 1997). The threat of cross-resistance to different antibiotics elevates the complexity of EPI discovery ventures.

The well-studied non-vertebrate hosts (the nematode *Caenorhabditis elegans*, the great wax moth *Galleria mellonella*, the fruit fly *Drosophilla melanogaster*, and the zebrafish *Danio rerio*) have been used to profile efflux based microbial virulence as well as to develop tractable, whole-animal antimicrobial screens (Apidianakis et al., 2007, 2011; Fuchs et al., 2010). *C. elegans* was used to assess the fitness of *in vitro* selected *Pseudomonas aeruginosa* MexAB-OprM (nalB) and MexCD-OprJ (nfxB) multidrug resistant mutants (Sánchez et al., 2002) and to confirm that overproduction of MexEF-OprN does not impair *P. aeruginosa* fitness in competition tests, but resulted in specific changes in bacterial regulatory networks (Olivares et al., 2012). *Burkholderia pseudomallei* can cause “disease-like” symptoms and kill the nematode but this killing mechanism is not related to efflux systems that pump out either aminoglycosides or macrolides (O'Quinn et al., 2001). A direct correlation between efflux mediated multidrug resistance and virulence was observed in *Klebsiella pneumoniae* when an array of antimicrobials was profiled in a *C. elegans* infection model (Bialek et al., 2010). Finally, the design of host-pathogen studies exploring the ability of efflux to interfere with virulence determinants appears promising but not informative, as observational results vary.

## Toward realistic efflux discovery tools

The need to protect a cell from amphipathic cations has evolved in different families of efflux systems across different organisms despite a lack of overall molecular homology or similarity in their mechanism of action. Thus, the RND super-family has a broad substrate spectrum, also found in ABC-transporters, including apart from antibiotics, amphipathic cations, biocides, dyes, and steroid hormones (Elkins and Nikaido, 2002; Lage, 2003; Elkins and Mullis, 2006).

Plants have been identified as sources of natural efflux substrates and inhibitors (Tegos, 2006). Disabling RNDs in plant and human bacterial pathogens led to a striking increase in antimicrobial activity (Tegos et al., 2002). As RNDs have a fundamental role in allowing bacteria to survive in their ecological niche, many host-derived compounds have been identified as potential substrates in humans, animals and plants (Piddock, 2006). In this context, it is important to highlight studies employing metabolomics to identify host-derived ABC efflux substrates in human fluids (Krumpochova et al., 2012; van de Wetering and Sapthu, 2012).

**Figure 1:**
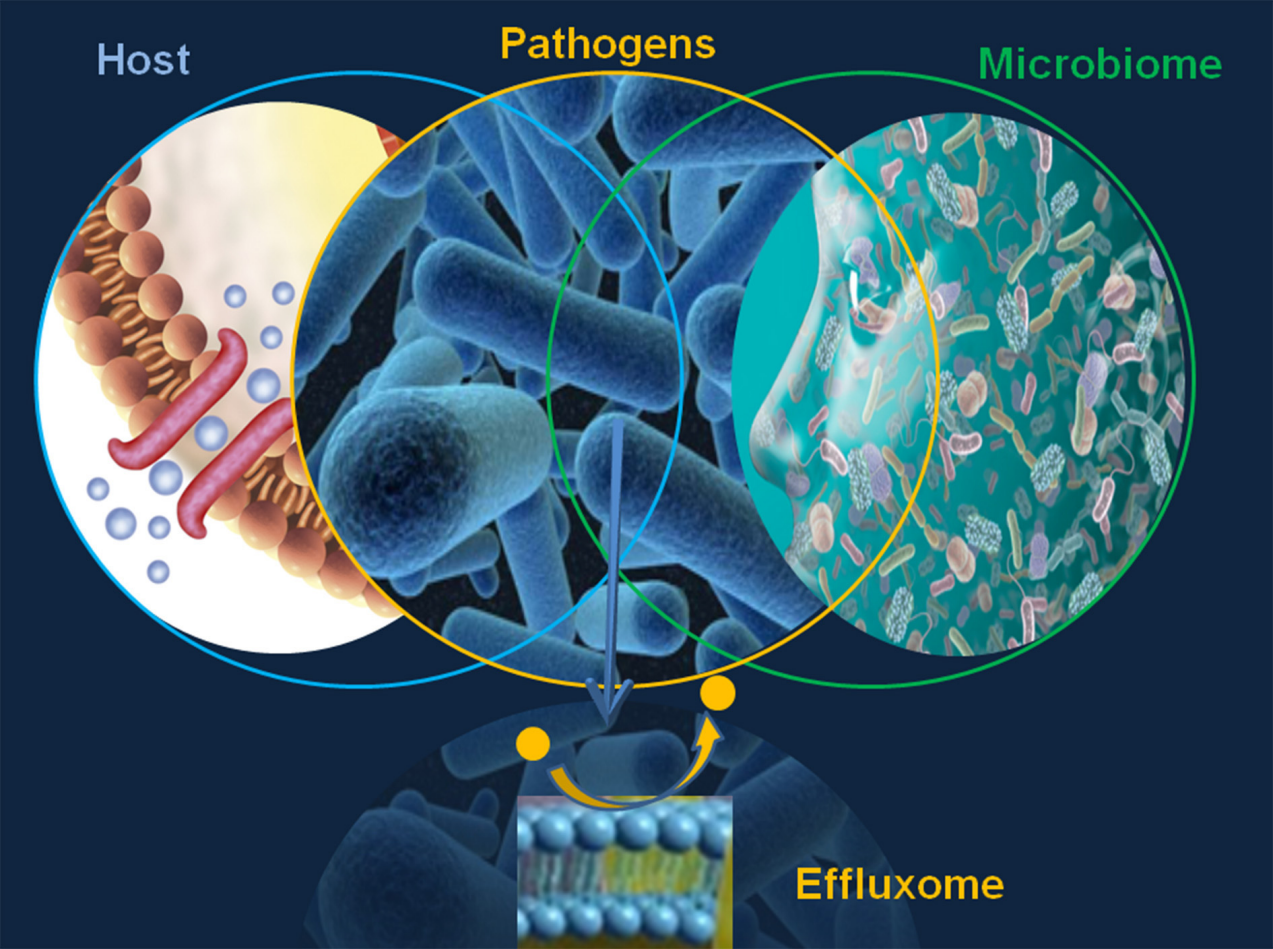
**The microbial effluxome**.

The major limitation in defining the microbial effluxome (the microbial efflux system substrate profile in context with the host physiology and pathology, Figure 1) is the elusive character of the “fingerprint” of the natural, host-derived microbial efflux substrates. This gap prevents any comprehensive discovery EPI effort and underlines the need for the design, validation and translation of highly informative efflux systems and substrate analyses.

Which factors will determine the effectiveness of efflux based anti-infective strategies? Are there any competitive benefits in the development of host-based instead of pathogen-based discovery applications? The systems-based computational bio-informatics and chemo-informatics tools appear as the appropriate stepping stone in the discovery process. Mapping of genomes and proteomes have been advancing at full speed, but without advanced mining and current laborious development it will not provide sufficient clarity for the importance of efflux systems in microbial networks behaviors, and identification of transporter roles within the context of the microbiome and infection.

Two currently available advanced efflux tools are (1) The ***TransportDB***, a comprehensive database of cytoplasmic membrane transporters and outer membrane channels in organisms with complete genome sequences (Ren et al., 2007). The database is annotated with functional transporter classification, web interfaces for easy access, query, and data download. Additionally, TransportDB allows comparative phylogenetic and substrate specificity analysis. (2) The ***Transporter-ligand interactome (TLI)*** is a knowledge mining tool built on the top of a chemo-informatics database that is used to collect, select, curate, organize, analyze, and build models as well as to distribute screening results and published bioactivity data related to fungal and mammalian ABC transporters. The TLI system provides the ability to interactively query and organize the collection of substrates, inhibitors, their associated assays and chemical structural features (Tegos et al., 2014).

### Conflict of interest statement

The authors declare that the research was conducted in the absence of any commercial or financial relationships that could be construed as a potential conflict of interest.

## References

[B1] Ahmed M. Borsch C. Neyfakh A. Schuldner S. (1993). Mutants of *Bacillus subtilis* multidrug transporter Bmr with altered sensitivity to the antihypertensive alkaloid reserpine. J. Biol. Chem. 268, 11086–11089. 8098708

[B2] Allen H. Looft T. Bayles D. O. Humphrey S. Levine U. Y. Alt D.. (2011). Antibiotics in feed induce prophages in swine fecal microbiomes. MBio 2:e00260-11. 10.1128/mBio.00260-11 22128350 PMC3225969

[B3] Allison K. Brynildsen M. P. Collins J. J. (2011). Metabolite-enabled eradication of bacterial persisters by aminoglycosides. Nature 473, 216–220. 10.1038/nature10069 21562562 PMC3145328

[B4] Andersson D. I. Hughes D. (2014). Microbiological effects of sublethal levels of antibiotics. Nat. Rev. Microbiol. 12, 465–478. 10.1038/nrmicro3270 24861036

[B5] Anyanful A. Dolan-Livengood J. M. Lewis T. Sheth S. Dezalia M. N. Sherman M. A.. (2005). Paralysis and killing of Caenorhabditis elegans by enteropathogenic *Escherichia coli* requires the bacterial tryptophanase gene. Mol. Microbiol. 57, 988–1007. 10.1111/j.1365-2958.2005.04739.x 16091039

[B6] Apidianakis Y. Mindrinos M. N. Xiao W. Tegos G. P. Papisov M. I. Hamblin M. R.. (2007). Involvement of skeletal muscle gene regulatory network in susceptibility to wound infection following trauma. PLoS ONE 2:e1356. 10.1371/journal.pone.0001356 18159239 PMC2131783

[B7] Apidianakis Y. Que Y.-A. Xu W. Tegos G. P. Zimniak P. Hamblin M. R.. (2011). Down-regulation of glutatione S-transferase alpha 4 (hGSTA4) in the muscle of thermally injured patients is indicative of susceptibility to bacterial infection. FASEB J. 26, 730–737. 10.1096/fj.11-192484 22038048 PMC3290433

[B8] Bialek S. Lavigne J. P. Chevalier J. Marcon E. Leflon-Guibout V. Davin A.. (2010). Membrane efflux and influx modulate both multidrug resistance and virulence of *Klebsiella pneumoniae* in a Caenorhabditis elegans model. Antimicrob. Agents Chemother. 54, 4373–4378. 10.1128/AAC.01607-09 20679507 PMC2944602

[B9] Brogden K. A. (2005). Antimicrobial peptides: pore formers or metabolic inhibitors in bacteria? Nat. Rev. Microbiol. 3, 238–250. 10.1038/nrmicro1098 15703760

[B10] Browning D. Wells T. J. França F. L. Morris F. C. Sevastsyanovich Y. R. Bryant J. A.. (2013). Laboratory adapted *Escherichia coli* K-12 becomes a pathogen of Caenorhabditis elegans upon restoration of O antigen biosynthesis. Mol. Microbiol. 87, 939–950. 10.1111/mmi.12144 23350972

[B11] Cohen N. Lobritz M. A. Collins J. J. (2013). Microbial persistence and the road to drug resistance. Cell Host Microbe. 13, 632–642. 10.1016/j.chom.2013.05.009 23768488 PMC3695397

[B12] Cryan J. F. Dinan T. G. (2012). Mind-altering microorganisms: the impact of the gut microbiota on brain and behaviour. Nat. Rev. Neurosci. 13, 701–712. 10.1038/nrn3346 22968153

[B13] Cunha B. A. (2001). Antibiotic side effects. Med. Clin. North Am. 85, 149–185. 10.1016/S0025-7125(05)70309-6 11190350

[B14] Dancer S. J. (2004). How antibiotics can make us sick: the less obvious adverse effects of antimicrobial chemotherapy. Lancet Infect. Dis. 4, 611–619. 10.1016/S1473-3099(04)01145-4 15451489

[B15] Dean M. Rzhetsky A. Allikmets R. (2001). The human ATP-binding cassette (ABC) transporter superfamily. Genome Res. 11, 1156–1166. 10.1101/gr.GR-1649R 11435397

[B16] Elkins C. A. Mullis L. B. (2006). Mammalian steroid hormones are substrates for the major RND- and MFS-type tripartite multidrug efflux pumps of *Escherichia coli*. J. Bacteriol. 188, 1191–1195. 10.1128/JB.188.3.1191-1195.2006 16428427 PMC1347360

[B17] Elkins C. A. Nikaido H. (2002). Substrate specificity of the RND-type multidrug efflux pumps AcrB and AcrD of *Escherichia coli* is determined predominantly by two large periplasmic loops. J. Bacteriol. 184, 6490–6498. 10.1128/JB.184.23.6490-6499.2002 12426336 PMC135441

[B18] Fletcher J. I. Haber M. Henderson M. J. Norris M. D. (2010). ABC transporters in cancer: more than just drug efflux pumps. Nat. Rev. Cancer 10, 147–156. 10.1038/nrc2789 20075923

[B19] Fuchs B. O'Brien E. Khoury J. B. Mylonakis E. (2010). Methods for using *Galleria mellonella* as a model host to study fungal pathogenesis. Virulence 1, 475–482. 10.4161/viru.1.6.12985 21178491

[B20] Guilhelmelli F. Vilela N. Albuquerque P. Derengowski Lda S. Silva-Pereira I. Kyaw C. M. (2013). Antibiotic development challenges: the various mechanisms of action of antimicrobial peptides and of bacterial resistance. Front. Microbiol. 4:353. 10.3389/fmicb.2013.00353 24367355 PMC3856679

[B21] Hassan K. Jackson S. M. Penesyan A. Patching S. G. Tetu S. G. Eijkelkamp B. A.. (2013). Transcriptomic and biochemical analyses identify a family of chlorhexidine efflux proteins. Proc. Natl. Acad. Sci. U.S.A. 110, 20254–20259. 10.1073/pnas.1317052110 24277845 PMC3864336

[B22] Hirakata Y. Kondo A. Hoshino K. Yano H. Arai K. Hirotani A.. (2009). Efflux pump inhibitors reduce the invasiveness of *Pseudomonas aeruginosa*. Int. J. Antimicrob. Agents 34, 343–346. 10.1016/j.ijantimicag.2009.06.007 19615866

[B23] Holzinger D. Gieldon L. Mysore V. Nippe N. Taxman D. J. Duncan J. A.. (2012). *Staphylococcus aureus* Panton-Valentine leukocidin induces an inflammatory response in human phagocytes via the NLRP3 inflammasome. J. Leukoc. Biol. 92, 1069–1081. 10.1189/jlb.0112014 22892107 PMC3476237

[B24] Hoskin D. W. Ramamoorthy A. (2008). Studies on anticancer activities of antimicrobial peptides. Biochim. Biophys. Acta 1778, 357–375. 10.1016/j.bbamem.2007.11.008 18078805 PMC2238813

[B25] Kazimierczak K. Rincon M. T. Patterson A. J. Martin J. C. Young P. Flint H. J.. (2008). A new tetracycline efflux gene, tet(40), is located in tandem with tet(O/32/O) in a human gut firmicute bacterium and in metagenomic library clones. Antimicrob. Agents Chemother. 52, 4001–4009. 10.1128/AAC.00308-08 18779355 PMC2573101

[B26] Klyachko K. Schuldiner S. Neyfakh A. A. (1997). Mutations affecting substrate specificity of the *Bacillus subtilis* multidrug transporter BMR. J. Bacteriol. 179, 2189–2193. 9079903 10.1128/jb.179.7.2189-2193.1997 PMC178954

[B27] Kourtesi C. Ball A. R. Huang Y. Y. Jachak S. M. Vera D. M. A. Khondkar P.. (2013). Microbial efflux systems and inhibitors: approaches to drug discovery and the challenge of clinical implementation. Open Microbiol. J. 7, 34–52. 10.2174/1874285801307010034 23569468 PMC3617545

[B28] Kraal L. Abubucker S. Kota K. Fischbach M. A. Mitreva M. (2014). The prevalence of species and strains in the human microbiome: a resource for experimental efforts. PLoS ONE 9:e97279. 10.1371/journal.pone.0097279 24827833 PMC4020798

[B29] Krumpochova P. Sapthu S. Brouwers J. F. de Haas M. de Vos R. Borst P.. (2012). Transportomics: screening for substrates of ABC transporters in body fluids using vesicular transport assays. FASEB J. 26, 738–747. 10.1096/fj.11-195743 22034653

[B30] Lage H. (2003). ABC-transporters: implications on drug resistance from microorganisms to human cancers. Int. J. Antimicrob. Agents 22, 188–199. 10.1016/S0924-8579(03)00203-6 13678820

[B31] Lemaire S. Van Bambeke F. Mingeot-Leclercq M. P. Glupczynski Y. Tulkens P. M. (2007). Role of acidic pH in the susceptibility of intraphagocytic methicillin-resistant *Staphylococcus aureus* strains to meropenem and cloxacillin. Antimicrob. Agents Chemother. 51, 1627–1632. 10.1128/AAC.01192-06 17307986 PMC1855560

[B32] Lomovskaya O. Bostian K. A. (2006). Practical applications and feasibility of efflux pump inhibitors in the clinic - a vision for applied use. Biochem. Pharmacol. 71, 910–918. 10.1016/j.bcp.2005.12.008 16427026

[B33] Martinez J. Sánchez M. B. Martínez-Solano L. Hernandez A. Garmendia L. Fajardo A.. (2009). Functional role of bacterial multidrug efflux pumps in microbial natural ecosystems. FEMS Microbiol. Rev. 33, 430–449. 10.1111/j.1574-6976.2008.00157.x 19207745

[B34] Merkx-Jacques A. Coors A. Brousseau R. Masson L. Mazza A. Tien Y. C.. (2013). Evaluating the pathogenic potential of environmental *Escherichia coli* by using the Caenorhabditis elegans infection model. Appl. Environ. Microbiol. 79, 2435–2445. 10.1128/AEM.03501-12 23377948 PMC3623224

[B35] Nguyen L. T. Haney E. F. Vogel H. J. (2011). The expanding scope of antimicrobial peptide structures and their modes of action. Trends Biotechnol. 29, 464–472. 10.1016/j.tibtech.2011.05.001 21680034

[B36] Nikaido H. (1996). Multidrug efflux pumps of gram-negative bacteria. J. Bacteriol. 178, 5853–5859. 8830678 10.1128/jb.178.20.5853-5859.1996 PMC178438

[B37] Olivares J. Alvarez-Ortega C. Linares J. F. Rojo F. Kohler T. Martinez J. L. (2012). Overproduction of the multidrug efflux pump MexEF-OprN does not impair *Pseudomonas aeruginosa* fitness in competition tests, but produces specific changes in bacterial regulatory networks. Environ. Microbiol. 14, 1968–1981. 10.1111/j.1462-2920.2012.02727.x 22417660

[B38] O'Quinn A. Wiegand E. M. Jeddeloh J. A. (2001). *Burkholderia pseudomallei* kills the nematode Caenorhabditis elegans using an endotoxin-mediated paralysis. Cell. Microbiol. 3, 381–393. 10.1046/j.1462-5822.2001.00118.x 11422081

[B39] Palmeira A. Sousa E. Vasconcelos M. H. Pinto M. M. (2012). Three decades of P-gp inhibitors: skimming through several generations and scaffolds. Curr. Med. Chem. 19, 1946–2025. 10.2174/092986712800167392 22257057

[B40] Peterson J. Garges S. Giovanni M. McInnes P. Wang L. Schloss J. A.. (2009). The NIH human microbiome project. Genome Res. 19, 2317–2323. 10.1101/gr.096651.109 19819907 PMC2792171

[B41] Piddock L. J. (2006). Multidrug-resistance efflux pumps - not just for resistance. Nat. Rev. Microbiol. 4, 629–636. 10.1038/nrmicro1464 16845433

[B42] Reddy K. V. Yedery R. D. Aranha C. (2004). Antimicrobial peptides: premises and promises. Int. J. Antimicrob. Agents 24, 536–547. 10.1016/j.ijantimicag.2004.09.005 15555874

[B43] Rees D. Johnson E. Lewinson O. (2009). ABC transporters: the power to change. Nat. Rev. Mol. Cell Biol. 10, 218–227. 10.1038/nrm2646 19234479 PMC2830722

[B44] Ren Q. Chen K. Paulsen I. T. (2007). TransportDB: a comprehensive database resource for cytoplasmic membrane transport systems and outer membrane channels. Nucleic Acids Res. 35, D274–D279. 10.1093/nar/gkl925 17135193 PMC1747178

[B45] Sánchez P. Linares J. F. Ruiz-Díez B. Campanario E. Navas A. Baquero F.. (2002). Fitness of *in vitro* selected *Pseudomonas aeruginosa* nalB and nfxB multidrug resistant mutants. J. Antimicrob. Chemother. 50, 657–664. 10.1093/jac/dkf185 12407121

[B46] Schloissnig S. Arumugam M. Sunagawa S. Mitreva M. Tap J. Zhu A.. (2013). Genomic variation landscape of the human gut microbiome. Nature 493, 45–50. 10.1038/nature11711 23222524 PMC3536929

[B47] Schneider D. S. Ayres J. S. (2008). Two ways to survive infection: what resistance and tolerance can teach us about treating infectious diseases. Nat. Rev. Immunol. 8, 889–895. 10.1038/nri2432 18927577 PMC4368196

[B48] Tan S. L. Ganji G. Paeper B. Proll S. Katze M. G. (2007). Systems biology and the host response to viral infection. Nat. Biotechnol. 25, 1383–1389. 10.1038/nbt1207-1383 18066032 PMC7097743

[B49] Tegos G. (2006). Substrates and inhibitors of microbial efflux pumps; redifine the role of plant antimicrobials, in Naturally Occurring Bioactive Compounds: a New and Safe Alternative for Control of Pests and Microbial Diseases, ed Mahendra Rai C. M. C. (Cambridge: Cambridge University Press), 45–55.

[B50] Tegos G. P. Evangelisti A. M. Strouse J. J. Ursu O. Bologa C. Sklar L. A. (2014). A high throughput flow cytometric assay platform targeting transporter inhibition. Drug Discov. Today Technol. 12, e95–e103. 10.1016/j.ddtec.2014.03.010 25027381 PMC4101541

[B51] Tegos G. Stermitz F. R. Lomovskaya O. Lewis K. (2002). Multidrug pump inhibitors uncover remarkable activity of plant antimicrobials. Antimicrob. Agents Chemother. 46, 3133–3141. 10.1128/AAC.46.10.3133-3141.2002 12234835 PMC128777

[B52] Tian B. Fadhil N. H. Powell J. E. Kwong W. K. Moran N. A. (2012). Long-term exposure to antibiotics has caused accumulation of resistance determinants in the gut microbiota of honeybees. MBio 3, 6. 10.1128/mBio.00377-12 23111871 PMC3487773

[B53] van de Wetering K. Sapthu S. (2012). ABCG2 functions as a general phytoestrogen sulfate transporter *in vivo*. FASEB J. 26, 4014–4024. 10.1096/fj.12-210039 22707564

[B54] Wexler H. (2012). Pump it up: occurrence and regulation of multi-drug efflux pumps in *Bacteroides fragilis*. Anaerobe 18, 200–208. 10.1016/j.anaerobe.2011.12.017 22266580

[B55] WHO (2014). Antimicrobial Resistance: Global Report on Surveillance. Geneva: World Health Organization.

[B56] Willenborg J. Willms D. Bertram R. Goethe R. Valentin-Weigand P. (2014). Characterization of multi-drug tolerant persister cells in *Streptococcus suis*. BMC Microbiol 14:120. 10.1186/1471-2180-14-120 24885389 PMC4040513

[B57] Wu Y. Vulić M. Keren I. Lewis K. (2012). Role of oxidative stress in persister tolerance. Antimicrob. Agents Chemother. 56, 4922–4926. 10.1128/AAC.00921-12 22777047 PMC3421885

[B58] Zeissig S. Blumberg R. S. (2014). Life at the beginning: perturbation of the microbiota by antibiotics in early life and its role in health and disease. Nat. Immunol. 15, 307–310. 10.1038/ni.2847 24646587

[B59] Zhang L. Kinkelaar D. Huang Y. Li Y. Li X. Wang H. H. (2011). Acquired antibiotic resistance: are we born with it? Appl. Environ. Microbiol. 77, 7134–7141. 10.1128/AEM.05087-11 21821748 PMC3194877

